# Non-ST-elevation myocardial infarction in the Netherlands: room for improvement!

**DOI:** 10.1007/s12471-020-01433-x

**Published:** 2020-06-03

**Authors:** P. Ten Have, A. D. Hilt, H. Paalvast, D. C. Eindhoven, M. J. Schalij, S. L. M. A. Beeres

**Affiliations:** 1 Zorginstituut Nederland, Diemen, The Netherlands; 2 Department of Cardiology, Leiden University Medical Centre, Leiden, The Netherlands

**Keywords:** Medication adherence, Myocardial infarction care, Non-ST-elevation myocardial infarction, National claims data

## Abstract

**Aim:**

To analyse non-ST-elevation myocardial infarction (NSTEMI) care in the Netherlands and to identify modifiable factors to improve NSTEMI healthcare.

**Methods:**

This retrospective cohort study analysed hospital and pharmacy claims data of all NSTEMI patients in the Netherlands in 2015. The effect of percutaneous coronary intervention (PCI) during hospitalisation on 1‑year mortality was investigated in the subcohort alive 4 days after NSTEMI. The effect of medical treatment on 1‑year mortality was assessed in the subcohort alive 30 days after NSTEMI. The effect of age, gender and co-morbidities was evaluated. PCI during hospitalisation was defined as PCI within 72 h after NSTEMI and optimal medical treatment was defined as the combined use of an aspirin species, P2Y_12_ inhibitor, statin, beta-blocker and angiotensin converting enzyme inhibitor/angiotensin II receptor blocker, started within 30 days after NSTEMI.

**Results:**

Data from 17,997 NSTEMI patients (age 69.6 (SD = 12.8) years, 64% male) were analysed. Of the patients alive 4 days after NSTEMI, 43% had a PCI during hospitalisation and 1‑year mortality was 10%. In the subcohort alive 30 days after NSTEMI, 47% of patients were receiving optimal medical treatment at 30 days and 1‑year mortality was 7%. PCI during hospitalisation (odds ratio (OR) 0.42; 95% confidence interval (CI) 0.37–0.48) and optimal medical treatment (OR 0.59; 95% CI 0.51–0.67) were associated with a lower 1‑year mortality.

**Conclusion:**

In Dutch NSTEMI patients, use of PCI during hospitalisation and prescription of optimal medical treatment are modest. As both are independently associated with a lower 1‑year mortality, this study provides direction on how to improve the quality of NSTEMI healthcare in the Netherlands.

**Electronic supplementary material:**

The online version of this article (10.1007/s12471-020-01433-x) contains supplementary material, which is available to authorized users.

## What’s new?

- European Society of Cardiology and American Heart Association guidelines emphasise the importance of assessing clinical performance indicators such as percutaneous coronary intervention (PCI) use and medication adherence in both ST-elevation myocardial infarction (STEMI) and non-STEMI (NSTEMI) patients.
- Insight into NSTEMI healthcare at a national level is crucial to improve survival in this large patient group, however not easily achieved.
- Healthcare claims data can be used to assess NSTEMI healthcare in a novel and accessible way.
- Dutch NSTEMI patient data show that utilisation of PCI and secondary prevention are suboptimal in this population.

## Introduction

Improvements in early recognition and revascularisation have significantly decreased mortality after myocardial infarction in recent decades [1–3]. This reduction in mortality, however, has been achieved especially in ST-elevation myocardial infarction (STEMI) patients. Unfortunately, the mortality rate in non-STEMI (NSTEMI) patients has not declined in recent years [4, 5].

The most recent European and American guidelines emphasise that the use of an early invasive strategy, as well as optimal medical therapy, contributes to a better long-term survival after NSTEMI (both evidence class 1, level A) [2, 3, 6]. The European guidelines even indicate them as performance measures of NSTEMI care, based on various large meta-analyses and randomised controlled trials [7, 8]. Recently, Hall et al. demonstrated that optimal use of guideline-indicated care for NSTEMI was associated with greater survival gain [9]. However, in Hall’s study the adherence rate was suboptimal, indicating that survival can potentially be improved. This recent study illustrates that large-scale monitoring of guideline-indicated care adherence is crucial to provide insight into how to improve NSTEMI care, ultimately resulting in improved survival.

The Dutch National Healthcare Institute (Zorginstituut Nederland, ZINL) advises the Dutch government with the primary objective of improving Dutch national healthcare. For this purpose, ZINL has access to all Dutch patients’ claims data in the Netherlands. The use of these claims data has been proven to reflect the real clinical data and to be correct and adequate in previous studies [10–12]. The current study is performed in close collaboration with ZINL and aims to analyse NSTEMI care in the Netherlands and to identify modifiable performance factors that can be used to analyse and improve the quality of NSTEMI healthcare in the Netherlands.

## Methods

**Fig. 1:**
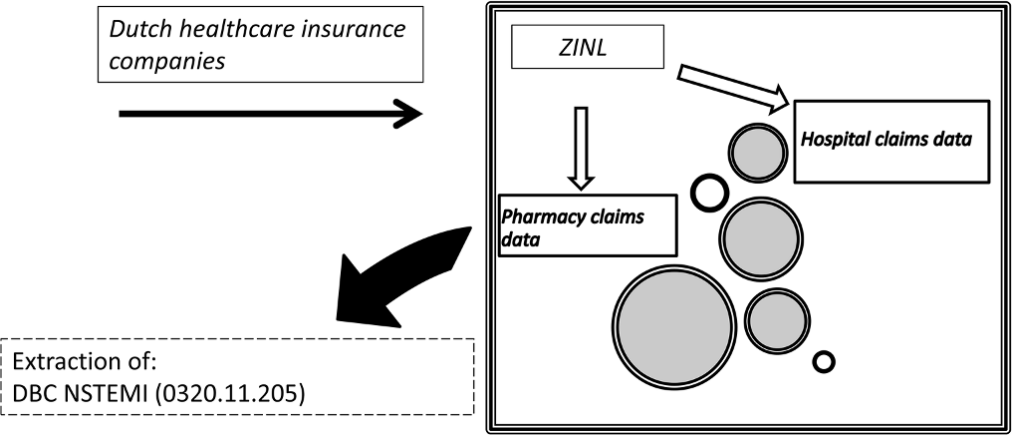
Data collection (*DBC* diagnosis treatment combination (*Diagnose Behandel Combinatie*), *NSTEMI* non-ST-elevation myocardial infarction, *ZINL* Dutch National Healthcare Institute)

Hospital claims are sent to patients’ insurance companies and subsequently collected in the central database of the insurance companies in the Netherlands. The use of this type of data has been validated in previous studies [11]. ZINL has access to both hospital and pharmacy claims databases and can access data at the patient level, which can be linked but is anonymous (Fig. 1).

### Study population

**Fig. 2:**
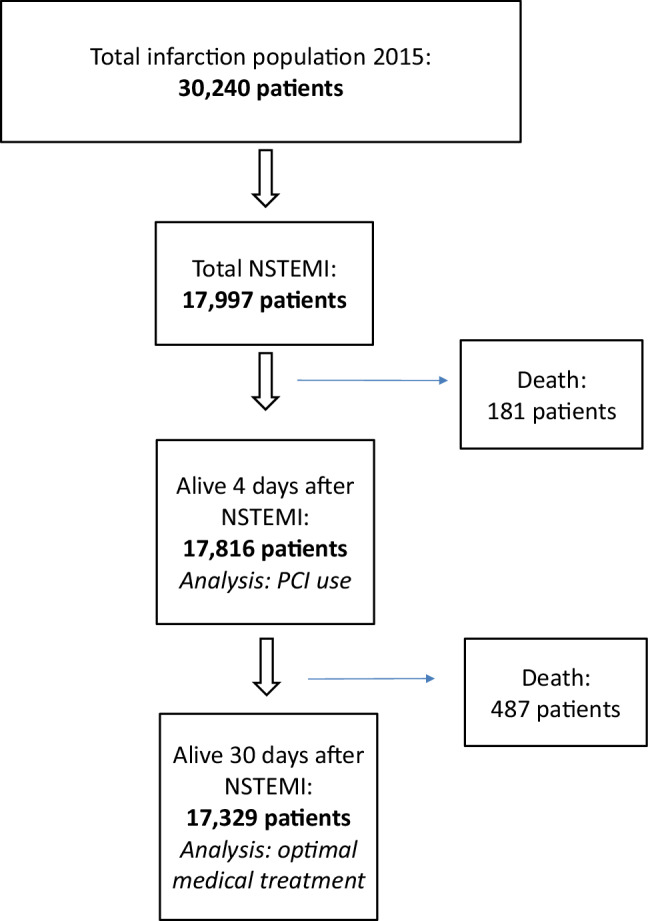
Non-ST-elevation myocardial infarction (*NSTEMI*) patient cohort. (*PCI* percutaneous coronary intervention. All healthcare claims from the Dutch healthcare insurance companies are stored and collected by the Dutch National Healthcare Institute (ZINL). This includes hospital claims data as well as pharmacy claims data. Data are available for analysis when approved (Fig. 1). From each dataset from different years, the claims for NSTEMI care can be collected for all patients (Fig. 2) but also for specific patient cohorts)

This is a retrospective cohort study. Three cohorts were used for analysis, as shown in Fig. 2.

- The entire study cohort comprises all Dutch patients above 18 years old who were admitted with a NSTEMI (diagnosis code 0320.11.205) in 2015. Only the first infarction during the study period was used for analysis. Patients who died on the 1st day of admission with the diagnosis of NSTEMI were excluded, in order to evaluate the effect of the different treatments.
- A subcohort, ‘NSTEMI‑4 days alive’, comprises all patients alive 4 days after NSTEMI. This subcohort was used to evaluate the effect of percutaneous coronary intervention (PCI) during hospitalisation on 1‑year mortality.
- A second subcohort, ‘NSTEMI-30 days alive’, comprises all patients alive 30 days after NSTEMI. This subcohort was used to evaluate the effect of medication use at 30 days on 1‑year mortality.

### Outcome measures: baseline characteristics

Age, gender, co-morbidities and 1‑year mortality were evaluated for all patients alive at 4 days and 30 days. For each patient, the absence or presence of diabetes mellitus, hypercholesterolaemia and obstructive pulmonary disease (COPD/asthma) at the time of infarction was determined based on medication use, 180 days before admission. Diabetes mellitus was defined as use of glucose-lowering medication (code A10XX, oral antidiabetics or insulin), hypercholesterolaemia as use of any form of cholesterol-lowering medication (code C10XX, all forms of cholesterol-lowering medication) and obstructive pulmonary disease as use of any inhalation medication (code R03XX, all forms of inhalation medication). These types of medication had to be used at least 180 days prior to the admission to qualify a patient as having the specified co-morbidity.

### PCI treatment

In the ‘NSTEMI‑4 days alive’ subcohort, the effect of PCI within 4 days of the diagnosis on mortality was evaluated (Fig. 2) in all patients alive at 4 days. The cut-off of 4 days was based on European and American guidelines, which recommend an invasive strategy, meaning coronary angiography, within the first 72 h, which is the acute or semi-acute phase of the infarction [6, 13]. PCI at a later time, for instance after 7 or 30 days, was not analysed as this was not deemed to be the ‘acute phase’ of the NSTEMI.

### Optimal medical treatment

In the ‘NSTEMI-30 days alive’ subcohort, the effect of complete optimal medical treatment on mortality was evaluated (Fig. 2). Optimal medical treatment after NSTEMI was defined as the combined use of an aspirin species (acetylsalicylic acid or carbasalate calcium), a P2Y_12_ inhibitor, a statin, a beta-blocker and an angiotensin converting enzyme (ACE) inhibitor/angiotensin II (AT2) receptor blocker. Fulfilled prescriptions of each of these were collected from the pharmacological database (GIP database).

### Statistical analysis

Data are presented as absolute numbers and as a proportion of the total population. Proportion comparisons were done by a χ^2^ test. Multivariate logistic regression analysis was done to understand the relation between 1‑year mortality as a dependent variable and independent variables: gender, age, diabetes, hypercholesterolaemia, obstructive pulmonary disease, use of PCI and optimal medical treatment. To assess the relation between treatment (PCI/optimal medical treatment) and 1‑year mortality, propensity score matching was applied.

### Statistics: propensity score matching

Patients were matched 1:1 using caliper matching. This procedure ensures an optimal balance of covariates between the treatment and the reference group. Age, gender and co-morbidities were analysed as risk factors and used for matching. When assessing the effectiveness of PCI and optimal medical treatment, interference of the added effect of the other (optimal medical treatment or PCI respectively when assessing the effect of optimal medical treatment) was unwanted and thus corrected for in all propensity score analyses. With propensity score matching a treatment and a reference cohort were created with a comparable load of risk factors. Matching was done specifically for PCI and a second time specifically for optimal medical treatment (Electronic Supplementary Material, Appendix A).

Statistical analysis was performed with R statistical program version 3.3.2 (x64), R packages MatchIt and tableone and SAS (SAS Institute, Cary, NC, USA.). For all tests, a *p*-value <0.05 was considered statistically significant.

### Ethical considerations

This research uses pseudo-anonymous and encrypted patient data. Dutch law states that prior ethical review and approval is not necessary.

## Results

### Study population

In 2015, a total of 30,240 myocardial infarction patients were admitted to Dutch hospitals, 60% being NSTEMI patients (*n* = 17,997). Their average age was 69.6 (SD = 12.8) years. Of the NSTEMI patients, 64% were male and 1‑year mortality was 11% (Tab. 1).

**Table 1:** Patient characteristics

	NSTEMI	NSTEMI 4 days	NSTEMI 30 days	*p*-value
Total patients (*n*)	17,997	17,816	17,329	
Age (average, SD)	69.6 (12.8)	69.5 (12.7)	69.2 (12.7)	NS
Male (*n*, %)	11,518 (64%)	11,388 (64%)	11,089 (64%)	NS
Diabetes (*n*, %)	3,779 (21%)	3,765 (21%)	3,610 (21%)	NS
Hypercholesterolaemia (*n*, %)	7,739 (43%)	7,632 (43%)	7,375 (43%)	NS
Obstructive pulmonary disease (*n*, %)	2,880 (16%)	2,793 (16%)	2,691 (16%)	NS
1‑year mortality	1,980 (11%)	1,781 (10%)	1,285 (7%)	<0.001

*NSTEMI* total non-ST-elevation myocardial infarction population, *NSTEMI 4 days* non-ST-elevation myocardial infarction patients alive at 4 days, *NSTEMI 30 days* non-ST-elevation myocardial infarction patients alive at 30 days

In the first 4 days 181 patients died. The subcohort ‘NSTEMI‑4 days alive’ consisted of 17,816 patients (Tab. 1, NSTEMI 4 days). Their average age was 69.5 (SD = 12.7) years with 64% being male, and 1‑year mortality in this cohort was 10%.

Additionally, in the following 26 days 487 patients died. This subcohort, ‘NSTEMI-30 days alive’, consisted of 17,329 patients (Tab. 1, NSTEMI 30 days). Their average age was 69.2 (SD = 12.7) years, with 64% being male. The 1‑year mortality in this cohort was 7%. Co-morbidities were frequent in all cohorts, and equally distributed (*p* = NS, Tab. 1).

### PCI treatment (NSTEMI-4 days alive)

In the ‘NSTEMI‑4 days alive’ subcohort (*n* = 17,816), PCI was performed within the first 72 h (3 days) in 43% of patients. Of interest, PCI was carried out in 35% of the female NSTEMI patients as compared to 47% of the male NSTEMI patients (*p* < 0.001).

### Medication use (NSTEMI-30 days alive)

Tab. 2 displays medication use at 30 days in the ‘NSTEMI-30 days alive’ subcohort (*n* = 17,329). In this subgroup, the percentage of patients with complete optimal medical treatment was 47% at 30 days. Use of aspirin species was 91%, of P2Y_12_ inhibitors 76%, of statins 85%, of beta-blockers 74% and of ACE inhibitors/AT2 receptor blockers 75%.

**Table 2:** Subcohort of non-ST-elevation myocardial infarction patients alive at 30 days: medication use at 30 days

Total complete optimal medical treatment use	8,144(47%)
Aspirin species	15,769(91%)
P2Y_12_ inhibitor	13,170(76%)
Statin	14,729(85%)
Beta-blocker	12,823(74%)
ACE inhibitor/AT2 receptor blocker	12,996(75%)

*ACE* angiotensin converting enzyme, *AT2* angiotensin II

### Effect of PCI on mortality (NSTEMI-4 days alive)

Tab. 3 displays the predictors of 1‑year mortality in the ‘NSTEMI‑4 days alive’ subcohort (*n* = 17,816). The following predictors add significantly to increased mortality: increasing age (odds ratio (OR) 1.09; 95% confidence interval (CI) 1.08–1.09), male gender (OR 1.27; 95% CI 1.14–1.42), diabetes mellitus (OR 1.51; 95% CI 1.34–1.70) and obstructive pulmonary disease (OR 1.52; 95% CI 1.37–1.71). Noticeably, PCI treatment within 4 days (OR 0.42; 95% CI 0.37–0.48) is associated with a substantially lower 1‑year mortality.

**Table 3:** Multivariate logistic regression of predictors of 1‑year mortality in the subcohort of non-ST-elevation myocardial infarction patients alive at 4 days

Factor	Odds ratio	95% confidence interval	*p*-value
Age (increase by 1 year)	1.09	1.08–1.09	<0.001
Male gender	1.27	1.14–1.42	<0.001
Diabetes mellitus	1.51	1.34–1.70	<0.001
Hypercholesterolaemia	1.11	0.99–1.23	NS
Obstructive pulmonary disease	1.52	1.37–1.71	<0.001
PCI during hospitalisation	0.42	0.37–0.48	<0.001

### Effect of medication on mortality (NSTEMI-30 days alive)

Predictors for 1‑year mortality in the ‘NSTEMI-30 days alive’ subcohort (*n* = 17,329) are shown in Tab. 4. In line with the entire study cohort, the following predictors add significantly to increased mortality: increasing age (OR 1.08; 95% CI 1.08–1.09), male gender (OR 1.21; 95% CI 1.07–1.37), diabetes mellitus (OR 1.54; 95% CI 1.34–1.76), hypercholesterolaemia (OR 1.23; 95% CI 1.08–1.39) and obstructive pulmonary disease (OR 1.61; 95% CI 1.40–1.85). Importantly, complete optimal medical treatment (OR 0.59; 95% CI 0.51–0.67) is associated with a substantially lower 1‑year mortality.

**Table 4:** Multivariate logistic regression of predictors of 1‑year mortality in the subcohort of non ST-elevation myocardial infarction patients alive at 30 days

Factor	Odds ratio	95% confidence interval	*p*-value
Age (increase by 1 year)	1.08	1.08–1.09	<0.001
Male gender	1.21	1.07–1.37	<0.01
Diabetes mellitus	1.54	1.34–1.76	<0.001
Hypercholesterolaemia	1.23	1.08–1.39	<0.01
Obstructive pulmonary disease	1.61	1.40–1.85	<0.001
Complete optimal medical treatment	0.59	0.51–0.67	<0.001
PCI during hospitalisation^a^	0.52	0.45–0.60	<0.001

*PCI* percutaneous coronary intervention

^a^In the patients alive at 30 days, the effect of PCI within 3 days was equally calculated to correct for it and to use this variable in propensity score matching (see Methods section)

### Propensity score matched cohort

Propensity score matching was performed in both cohorts; 14,364 patients could be matched in the case of PCI and 13,038 patients in the case of optimal medical treatment. After matching, the standardised mean differences of nearly all covariates were less than 0.1. The effect of PCI and the effect of optimal medical treatment, when compared with a reference group with a nearly identical mix of covariates, was significant (*p* < 0.001), stressing that both PCI within 4 days and optimal medical treatment are both significantly associated with increased survival in the Dutch NSTEMI population (Electronic Supplementary Material, Appendix A).

## Discussion

The current study analyses NSTEMI care in the Netherlands through hospital and pharmacy claims data. The main findings can be summarised as follows: NSTEMI patients are predominantly older, male patients. The use of PCI within 4 days of hospitalisation and the prescription of complete optimal medication treatment within 30 days after NSTEMI are both modest, but both significantly reduce 1‑year mortality in these patients. Non-use of both factors is independently associated with increased mortality, which suggests that through the present study NSTEMI healthcare quality can be improved in the Netherlands.

The current call for awareness of a wider use of PCI within 4 days of admission after NSTEMI and of optimal medical treatment prescription is in line with the recommendations in the European Society of Cardiology guidelines [3]. Furthermore, it is congruent with a recently published study by Hall et al. analysing over 400,000 hospital survivors of a NSTEMI in England and Wales, in order to investigate whether improved survival is associated with the use of NSTEMI guideline-indicated treatments [9]. In this somewhat older cohort (2003–2013), Hall et al. demonstrate that guideline-indicated treatment is associated with improved survival that persisted over the longer term. An invasive coronary strategy was found to have the most comprehensive and persistent impact on survival. This is consistent with the finding of the current study that PCI treatment for NSTEMI substantially reduces 1‑year mortality with an OR of 0.42.

A potential argument for non-use of PCI in NSTEMI patients in daily practice may be that the beneficial effect of PCI is less in the elderly with more co-morbidities, and complications are more prominent in this group. Interestingly, Couture et al. found that especially in older patients with more co-morbidities a more invasive strategy should be considered [14]. With the NSTEMI cohort in our study having a similar profile and low PCI rate, this can be an important finding indicating that there is room for improvement in the Netherlands. This issue was indeed also raised in a very recent registry by Hoedemaker et al., who evaluated treatment patterns of NSTEMI patients in 23 non-PCI centres in the Netherlands. In this registry, the majority of high-risk patients underwent angiography at a non-PCI centre. Despite the guideline recommendation only a quarter of these high-risk patients were transferred to a PCI centre within 1 day [15].

Apart from early PCI, the present study also underlines the importance of implementing optimal medical treatment within 30 days after NSTEMI. Whereas Hall et al. reported on recipes at discharge, the current study reveals which medication is collected at pharmacies up to 30 days after NSTEMI. Since primary non-adherence to prescribed medication has been reported to be very common in patients with ischaemic heart disease [16], we think that the current results provide additional insight into the significance of secondary prevention medication use after NSTEMI, especially since mortality differences were already observed within the 1st year. Accordingly, a conjoint effort of cardiologists and patients is warranted to improve medication adherence after NSTEMI.

The current study confirms the findings of Hall et al. and extends them to the entire Dutch population with additional insights into the impact of secondary prevention medication truly collected at pharmacies. Interestingly, the method of data collection substantially differs between the two studies. In particular, Hall et al. reported on data gathered from national clinical audits. Data collection for audits, however, is time consuming and expensive. We want to stress the need for new effective ways of evaluating healthcare as a key element in healthcare innovation. The use of claims data has been proven to be correct and adequate in previous studies [10, 11]. National claims data provide a good representation of the ‘real world’ setting in contrast to the common single-centre registries in NSTEMI studies [17–22]. Furthermore, such data have the following advantages: low administrative load, low costs, absence of reporting bias, and easy follow-up of patients being treated in more than one hospital.

Some limitations should nonetheless be considered when interpreting the results. First, the study uses a non-randomised design with observational data. The addition of propensity score matching, however, strengthens the results [23]. Second, this study assessed PCI treatment only in the acute phase of the infarction; an effect of PCI on mortality after the acute phase of NSTEMI was not calculated. Likewise, the effect of bypass surgery on mortality after NSTEMI was not calculated. Third, the level of clinical detail is limited, e.g. completeness of revascularisation or infarct size. Commonly used risk scores (e.g. Global Registry of Acute Coronary Events (GRACE) risk score) cannot be applied either. Potentially there could have been a bias from patients who were registered as NSTEMI but did not meet all the criteria of myocardial infarction and therefore should have been classified as having unstable angina. For our study, however, we rely on correct registration by Dutch cardiologists. Equally, the definitions used for co-morbidities are only determined through medication use prior to the infarction and not via clinical data. Likewise, clinical details on differences between male and female PCI rates are lacking in financial claims data. Fourth, although mortality data are available, the cause of death is not specified. Fifth, the pharmacy claims data only represent the collected medication, not the consumed medication. Furthermore, it remains unclear if medication was contra-indicated or not prescribed. And lastly, further randomised trials are needed to validate the healthcare benefit of PCI during hospitalisation and optimal medical treatment.

### Future perspectives

The use of financial claims data in the medical field is a relatively novel and modern way of analysing healthcare. It provides insight into where healthcare quality can be improved, both for clinicians and patients as well as for healthcare managers, insurance companies and policy makers at a national level. Particular attention should be given to a wider use of PCI within 4 days of admission for NSTEMI as well as to patient, doctor and financial factors contributing to medication adherence. Financial claims data can again be used to monitor the impact of such initiatives.

## Conclusion

The present study analysed hospital and pharmacy claims data of more than 17,000 NSTEMI patients in the Netherlands. PCI use during hospitalisation and optimal medical treatment are both moderately applied in this patient group but are independently associated with a lower 1‑year mortality. These findings importantly suggest that attention to a wider use of PCI during hospitalisation as well as particular attention to optimal medical treatment prescription by cardiologists and medication adherence by patients may substantially improve outcome after NSTEMI.

## Caption Electronic Supplementary Material

APPENDIX A: propensity score matching for PCI during hospitalisation and complete optimal medical treatment-use
