# Supplementary material for: Non-ST-elevation myocardial infarction in the Netherlands: room for improvement!
Source: Neth Heart J. 2020 Jun 3;28(10):537–45. doi: 10.1007/s12471-020-01433-x (PMC7494715; doi:10.1007/s12471-020-01433-x)
Supplement: Supplementary file 1 — APPENDIX A: propensity score matching for PCI during hospitalisation and complete optimal medical treatment-use [file 12471_2020_1433_MOESM1_ESM.docx]

**APPENDIX A: propensity score matching for PCI during hospitalisation and complete optimal medical treatment-use.**

**Part 1: PCI**

To assess the effect of PCI during hospitalisation on 1-year mortality, a treatment (PCI) and a reference group with an identical balance of covariates were created. This was done by calculating propensity scores with the R programming language and logistic regression on covariates: age, gender, use of diabetes mellitus medication in year before NSTEMI, use of medication for obstructive pulmonary disease in the year before NSTEMI and use of medication for hypercholesterolemia in year before NSTEMI.

With the R package ‘MatchIt’ patients were matched 1:1 based on corresponding propensity scores and a caliper of 0.9. Of the patients, 14,368 were matched. After matching the histograms of propensity scores of optimal medical treatment and reference group were nearly identical.


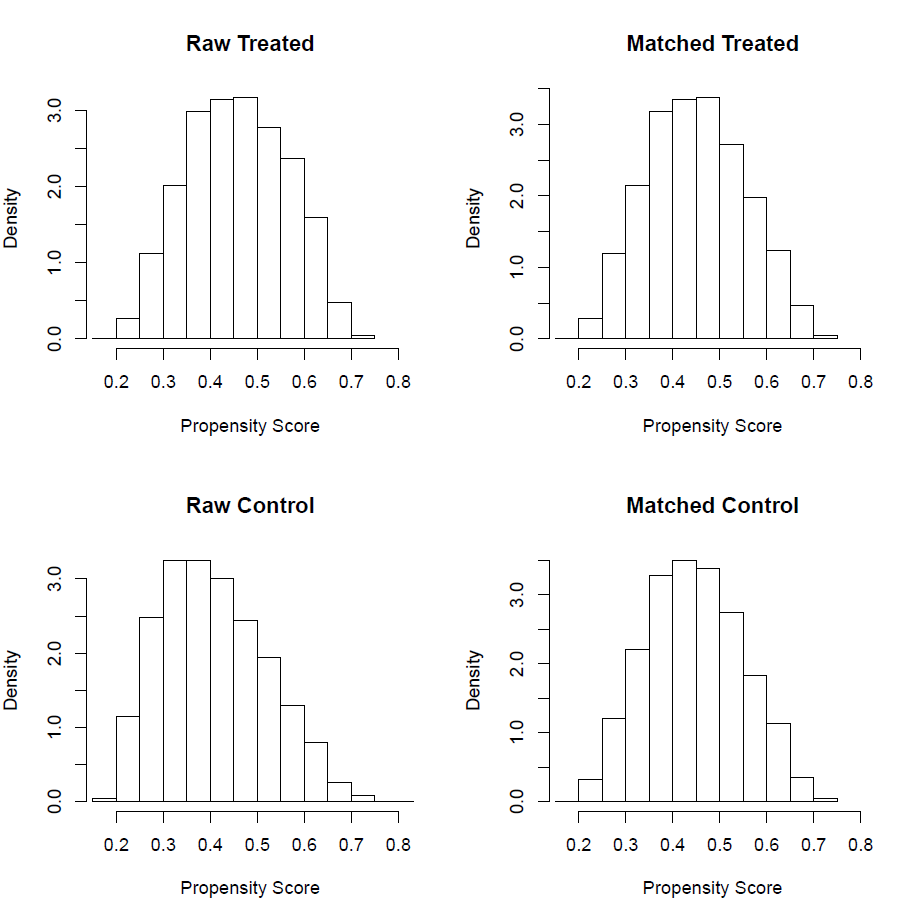


With the R package ‘tableone’ we checked if standardized mean differences (SMD) between the PCI and the reference group were comparable. Before propensity score matching (unadjusted table), age and sex were unevenly distributed between the reference and PCI group. After propensity score matching (adjusted table), all variables were evenly distributed; nearly all SMDs were < 0.1.

| Unadjusted (before propensity scoring) | | | Reference Group | PCI group | SMD |
| --- | --- | --- | --- | --- | --- |
| Patients (N=) |  |  | 10,172 | 7,644 |  |
| Age (Mean (SD)) |  |  | 71.69(12.91) | 66.48(11.89) | 0.420 |
| Male (Mean(SD)) |  |  | 0.59(0.49) | 0.71(0.46) | 0.243 |
| Diabetes Mellitus (Mean(SD) | |  | 0.23(0.42) | 0.19(0.39) | 0.109 |
| Hypercholesterolemia (Mean(SD)) | |  | 0.46(0.50) | 0.39(0.49) | 0.129 |
| Obstructive Pulmonary Disease (Mean(SD)) | | | 0.18(0.38) | 0.13(0.34) | 0.121 |

| Adjusted (after propensity scoring) | | | Reference Group | PCI group | SMD |
| --- | --- | --- | --- | --- | --- |
| Patients (N=) |  |  | 7,184 | 7,184 |  |
| Age (Mean (SD)) |  |  | 67.87 (12.29) | 67.54 (11.40) | 0.028 |
| Male (Mean(SD)) |  |  | 0.69 (0.46) | 0.70 (0.46) | 0.021 |
| Diabetes Mellitus (Mean(SD) | |  | 0.20 (0.40) | 0.19 (0.39) | 0.034 |
| Hypercholesterolemia (Mean(SD)) | |  | 0.42 (0.49) | 0.41 (0.49) | 0.018 |
| Obstructive Pulmonary Disease (Mean(SD)) | | | 0.14 (0.35) | 0.13 (0.34) | 0.026 |

With a paired T test we evaluated the effect difference on 1-year mortality. The effect difference of PCI treatment was significant (P < 0.001). Effect size: In this total group of 14,368 matched patients, the 1-year mortality was 7.7%. In the PCI group 1-year mortality was 4.6%, in the reference group 1-year mortality was 10.9%.

**Part 2: Complete optimal medical treatment (OMT)**

To assess the effect of complete optimal medical treatment on 1-year mortality, a treatment (optimal medical treatment) and a reference group with an identical balance of covariates were created. This was done by calculating propensity scores with the R programming language and logistic regression on covariates: age, gender, use of diabetes mellitus medication in year before NSTEMI, use of medication for obstructive pulmonary disease in the year before NSTEMI, use of medication for hypercholesterolemia in year before NSTEMI and PCI during hospitalisation.

With the R package ‘MatchIt’ patients were matched 1:1 based on corresponding propensity scores and a caliper of 0.5. Of the patients, 13,456 were matched. After matching the histograms of propensity scores of (optimal medical treatment) treatment and reference group were nearly identical.


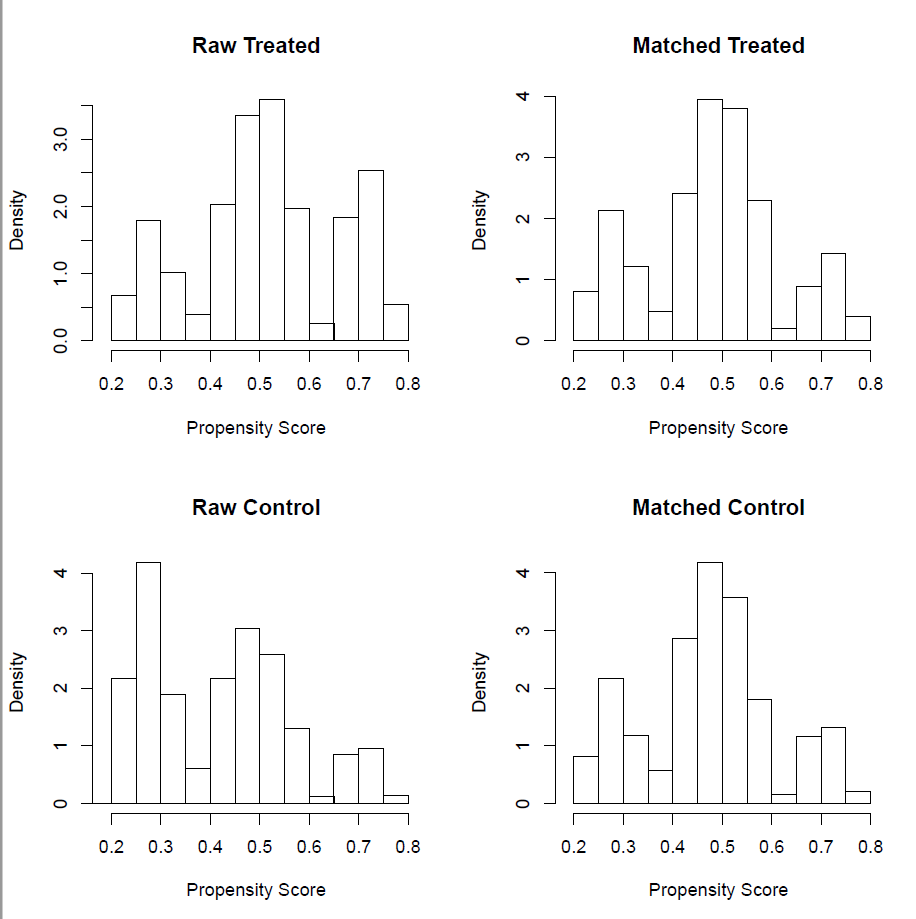


With the R package ‘tableone’ we checked if standardized mean differences (SMD) between the optimal medical treatment and the reference group were comparable. Before propensity score matching, noticeably patients with hypercholesterolemia and having had a PCI during hospitalisation were unevenly distributed amongst the reference and OMT group. After propensity score matching, nearly all SMDs for used variables were < 0.1.

| Unadjusted (before propensity scoring) | | | Reference Group | OMT group | SMD |
| --- | --- | --- | --- | --- | --- |
| Patients (N=) |  |  | 9,303 | 8,026 |  |
| Age (Mean (SD)) |  |  | 70,07(13.03) | 68.11(12.23) | 0.156 |
| Male (Mean(SD)) |  |  | 0.62(0.49) | 0.67(0.47) | 0.102 |
| Diabetes Mellitus (Mean(SD) | |  | 0.18(0.39) | 0.24(0.43) | 0.137 |
| Hypercholesterolemia (Mean(SD)) | |  | 0.35(0.48) | 0.51(0.50) | 0.323 |
| Obstructive Pulmonary Disease (Mean(SD)) | | | 0.16(0.37) | 0.15(0.36) | 0.036 |
| PCI during hospitalisation (Mean(SD)) | | | 0.33(0.47) | 0.57(0.50) | 0.497 |

| Adjusted (after propensity scoring) | | | Reference Group | OMT group | SMD |
| --- | --- | --- | --- | --- | --- |
| Patients (N=) |  |  | 6,728 | 6,728 |  |
| Age (Mean (SD)) |  |  | 69.32 (12.40) | 68.01 (12.59) | 0.105 |
| Male (Mean(SD)) |  |  | 0.64 (0.48) | 0.65 (0.48) | 0.004 |
| Diabetes Mellitus (Mean(SD) | |  | 0.21 (0.41) | 0.23 (0.42) | 0.054 |
| Hypercholesterolemia (Mean(SD)) | |  | 0.47 (0.50) | 0.44 (0.50) | 0.068 |
| Obstructive Pulmonary Disease (Mean(SD)) | | | 0.16 (0.36) | 0.16 (0.37) | 0.011 |
| PCI during hospitalisation (Mean(SD)) | | | 0.45 (0.50) | 0.49 (0.50) | 0.075 |

With a paired T test we evaluated the effect difference on 1-year mortality. The effect difference of optimal medical treatment was significant (P < 0.001). Effect size: In the total group of 13,456 matched patients, the 1-year mortality was 6.7%. In the optimal medical treatment group it was 5.1% and in the reference group it was 8.6%.
